# Quantification and composition of pharmaceutical waste in New Zealand

**DOI:** 10.1007/s10163-022-01410-z

**Published:** 2022-05-10

**Authors:** Sara M. Hanning, Changji Hua, Saeid Baroutian, Rob Burrell, Matthew Taylor, L. James Wright, Darren Svirskis

**Affiliations:** 1 School of Pharmacy, Faculty of Medical and Health Sciences, University of Auckland, 85 Park Road, Grafton, Auckland, 1023 New Zealand; 2 Department of Chemical and Materials Engineering, Faculty of Engineering, University of Auckland, Auckland, New Zealand; 3 Department of Anaesthesia, Middlemore Hospital, Counties Manukau Health, Private Bag 93311, Otahuhu, Auckland, 1640 New Zealand; 4 Department of Anaesthesiology, Faculty of Medical and Health Sciences, University of Auckland, 85 Park Road, Grafton, Auckland, 1023 New Zealand; 5 School of Chemical Sciences, The University of Auckland, Auckland, 1023 New Zealand

**Keywords:** Pharmaceutical waste, Waste production, Waste stream composition

## Abstract

This study aimed to quantify the amount of pharmaceutical waste produced in New Zealand, and determine the composition of pharmaceutical waste from community pharmacies in Auckland, New Zealand. Pharmaceutical waste collected in New Zealand is increasing, peaking at 542 tonne in 2019. Pharmaceutical waste collected from hospitals and pharmacies in Auckland increased by more than fourfold from 2016 to 2020. An audit of the types of pharmaceutical waste collected from community pharmacies revealed that the most common classes of drugs identified in this waste stream belonged to the nervous system, cardiovascular system and alimentary tract, and metabolism. Following examination of the contents of 12 pharmaceutical waste bins, 475 different pharmaceutical products were identified, highlighting the breadth of drugs in this waste stream. A range of dosage forms and hence materials were identified, which could present challenges for future waste treatment approaches. Hazardous drugs were identified including cytotoxic compounds, which should go into a separate waste stream for incineration. There is a need for similar data to be collected from multiple sites to fully appreciate the magnitude and composition of pharmaceutical waste. This will allow for the suitability of current practices for managing this hazardous waste stream to be evaluated.

## Introduction

Pharmaceutical waste is non-infectious medical waste. It includes medicines or items that may be contaminated by medicines such as associated packaging, medical devices, or in some cases, personal protective equipment (PPE) that is contaminated with medicines, but not infectious agents. Pharmaceutical waste is generated from a variety of sources including hospitals, private clinics, community pharmacies, the pharmaceutical industry, laboratories and research centres, and individual households [1].

Pharmaceutical waste generated from hospital and community pharmacies may include patient returned medicines or expired products. The quantification of pharmaceutical waste assumes that it is entering the correct waste stream. For example, individual households generate pharmaceutical waste, but this is often treated with general household waste. In many countries, individuals are encouraged to return unwanted or expired pharmaceutical products to pharmacies, who pass them on for disposal. In Australia, the public are encouraged to return pharmaceutical waste to community pharmacies, in a government-funded initiative called the National Return and Disposal of Unwanted Medicines (RUM) scheme [2]. Medicine collected in specific RUM bins is disposed of by high-temperature incineration as approved by the Environment Protection Authority [3]. As of February 2022, over 11,405 tonnes of medicines have been collected and incinerated in Australia since the inception of the RUM scheme in 1998 [2]. Similar programmes are available in other countries including Sweden and the United Kingdom. In some regions, mixing waste streams is standard practice. The US Food and Drug Administration (FDA) website includes recommendations for disposing of medicines at home, including flushing down the toilet or placing in a container and adding to general household waste [3], despite this practice being discouraged by the Environmental Protection Agency (EPA) [4]. In New Zealand, people are encouraged to return their unwanted medicines to their pharmacy for safe disposal and all pharmacies offer a free-to-consumer collection and disposal service for unwanted medicines [5]. However, according to a New Zealand survey of 452 participants, only 13–24% of people return their pharmaceutical waste to pharmacies depending on formulation, with capsules and tablets being more likely returned and liquid formulations less likely returned [6]. Even when medicines are correctly returned to pharmacies, they may still end up in an incorrect processing stream. One study found that when returned to pharmacies, 52.1% of liquids and over 73.3% of Class B controlled drugs were either poured down the sink or flushed down the toilet [7]. This means that they enter sewage, which are then treated in facilities not designed for treating pharmaceutical waste, before being discharged into the environment. Comparing sewage before and after treatment in a wastewater treatment plant in New Zealand showed that less than half of drugs like trimethoprim and metoprolol are removed [8].

**Fig. 1:**
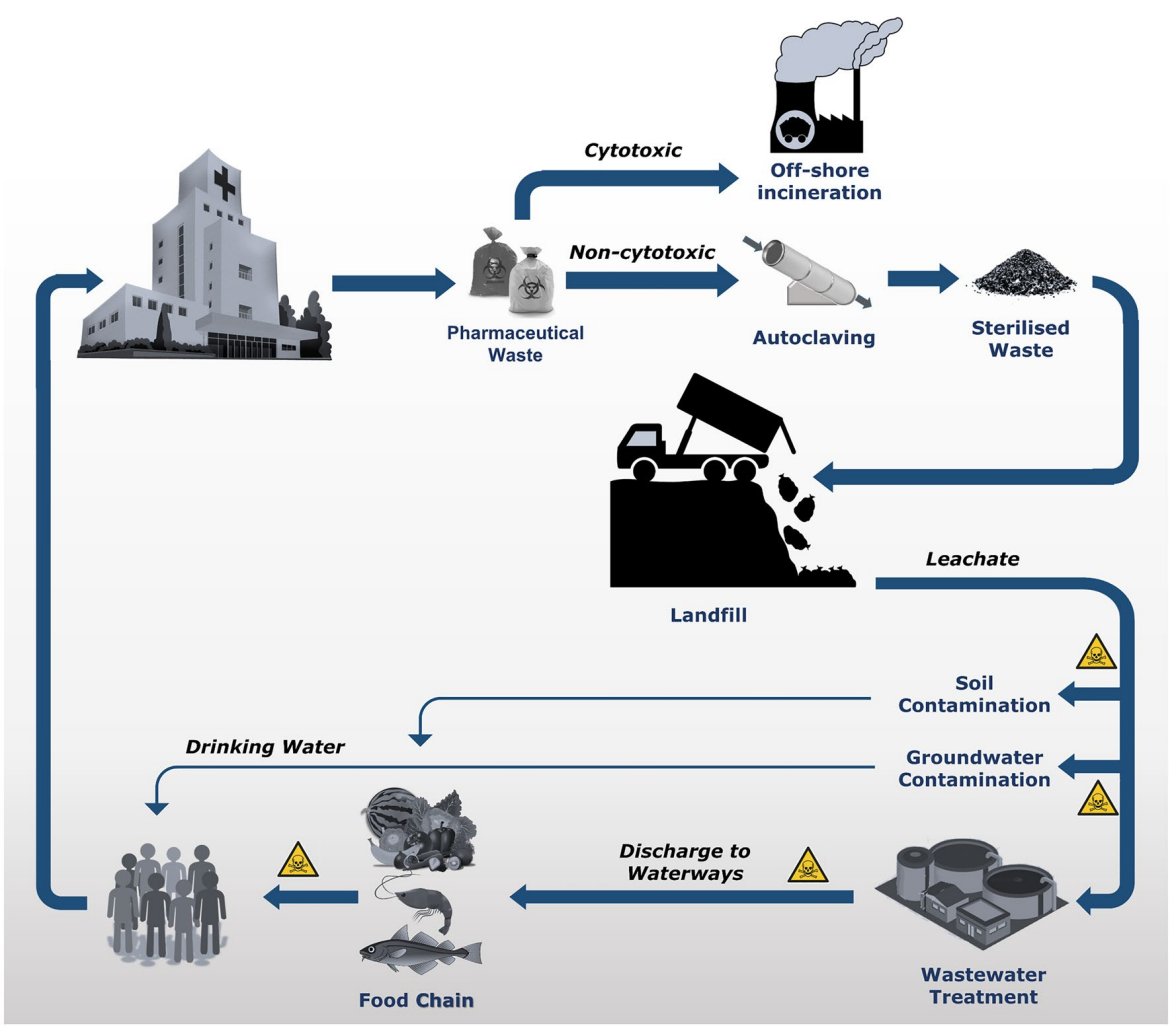
Overview of the pharmaceutical waste disposal process in New Zealand. Pharmaceutical waste from hospitals and pharmacies comprises expired medicines as well as unwanted medicines, particularly those returned from patients. Cytotoxic waste is sent off-shore for incineration, whereas other pharmaceutical waste is autoclaved before being sent to landfill. Individual images obtained from Pixabay

The pharmaceutical waste disposal process in New Zealand is depicted in Fig. 1. The pharmaceutical waste stream is autoclaved at 130 °C for approximately 30 min, before joining the general waste disposal stream and ending up in landfills. However, autoclaving is not designed to deactivate chemical or pharmaceutical waste, merely decontaminate it [9]. Therefore, even after this treatment, pharmaceutical contaminants can still enter the environment unaltered through leachate after the treated waste has reached landfill. It is illegal to operate high-temperature hazardous waste incinerators in New Zealand [10]. Paradoxically, cytotoxic waste is a highly hazardous subtype of pharmaceutical waste that in New Zealand is required to be incinerated at high temperatures under New Zealand Standard (NZS) 4304 [11]. Therefore, all cytotoxic waste is exported for incineration [12]. Export and incineration of cytotoxics in a way that is environmentally acceptable and satisfies the Basel convention on the international movement of hazardous waste [13] is expensive and increases the carbon footprint of cytotoxic pharmaceuticals.

Pharmaceutical waste can cause immediate harm to those who handle it and can also cause cumulative damage by contaminating the environment [14]. However, little is known about how much pharmaceutical waste is generated, and what the composition of this waste is. This report quantifies the amount of pharmaceutical waste produced by public hospitals, clinics, and pharmacies in Auckland, New Zealand. Auckland is the most populous city in New Zealand, with a population of 1.66 million, and makes up 34.6% of the population in New Zealand as of 2017 [15]. In addition, this report provides a snapshot of the specific types of pharmaceutical waste that are being disposed of from community pharmacies in the Auckland region.

## Materials and methods

### Quantification of pharmaceutical waste

Pharmaceutical waste was quantified with data provided by Interwaste New Zealand, a business contracted to provide waste disposal services to healthcare facilities across New Zealand. Interwaste New Zealand has exclusive contracts to collect waste from public hospitals and community pharmacies within the three District Health Boards (DHBs) in the Auckland region: Auckland DHB, Counties Manukau DHB, and Waitemata DHB.

Data about the quantity of pharmaceutical waste interwaste collected from DHB-contracted pharmacies and hospitals in Auckland from January 2016 to December 2020 were obtained from records held at Interwaste. For each DHB-contracted pharmacy and hospital over this time, the weight of collected waste, number of bins of waste collected, and whether or not the collected waste bins were labelled as containing cytotoxic products were determined. Trends over time were analysed.

### Composition of pharmaceutical waste

To gain a snapshot of the types of pharmaceutical waste generated by Auckland’s community pharmacies, 12 site visits to Interwaste took place between 6 January and 4 February 2021. On each visit, a random 120 L bin containing waste from an Auckland community pharmacy was opened and examined. The name, strength/concentration, and formulation of each medicine was recorded. Information such as whether or not the medicine was expired, and whether or not it contained identifying information was also noted. The number of units of each medicine found during each visit was recorded. One unit was defined as one tablet or capsule for solid dosage forms, and one original tube or one original bottle for semi-solid and liquid dosage forms.

To determine loose tablets and capsules that were not immediately identifiable, photographs were used to perform an online search to identify the medicine. A reverse search on the suspected medicine’s data sheet was also performed to ensure accuracy.

All collected data were sorted and analysed. Each medicine was then assigned a classification status, and a therapeutic group according to the World Health Organisation Anatomical Therapeutic Classification (ATC) index [16]. The number of times a particular drug appeared over the 12 visits was calculated.

## Results and discussion

### Quantification of pharmaceutical waste

The total pharmaceutical waste collected by Interwaste across New Zealand increased from 381 tonnes in 2016 to 542 tonnes in 2019, before decreasing slightly to 531 tonne in 2020. Auckland’s population makes up approximately 34.6% of New Zealand’s population [15]; however, 75.1% of national pharmaceutical waste produced between 2016 and 2020 was from the Auckland region. This likely reflects the fact that the majority of pharmaceutical companies and several research institutes are located in Auckland. The contribution of pharmacies and public hospitals in Auckland to the total weight of pharmaceutical waste collected by Interwaste across New Zealand between January 2016 and December 2020 increased from 2.2 to 8.8% (Table 1), a fourfold increase yet still a minor contribution to the overall production of pharmaceutical waste.

**Table 1:** Total weight of pharmaceutical waste collected from Interwaste in New Zealand, Auckland, and Auckland DHB-contracted services (hospitals and community pharmacies in the metropolitan Auckland regions) between 2016 and 2020

Year	Pharmaceutical waste collected in NZ (kg)	Total pharmaceutical waste collected in Auckland (%)	Total pharmaceutical waste collected from DHB-contracted services in Auckland (%)
2016	380,606	76.9	2.8
2017	423,698	77.1	4.9
2018	463,892	73.8	5.7
2019	541,653	75.2	5.9
2020	531,062	73.4	8.8

Over the last 4 years, there has been an increase in the weight of pharmaceutical waste being collected from pharmacies and hospitals in Auckland, as shown in Table 1. This increase may be in part the result of improvements to the monitoring of waste volumes over the past 5 years as a result of funding of the service at a DHB level. There may also be more awareness around the appropriate disposal of waste, leading to an increase in visibility of pharmaceutical waste as more is being directed into the correct waste stream. Further investigation into pharmaceutical waste entering other waste streams over time is needed to support this hypothesis.

Focusing on more recent data, the total waste production in Auckland compared to the rest of New Zealand between 2019 and 2020 increased markedly compared with previous years. This may be related to the COVID-19 pandemic response, as the increased use and disposal of PPE in hospitals and quarantine services such as gloves, facemasks, and overalls all contribute to waste generation. While this should be included in the medical waste stream, it is possible that some goes into the pharmaceutical waste stream, especially in community pharmacies where there is no dedicated medical waste stream. As of December 2020, 43% of all community COVID-19 cases in New Zealand were in the Auckland region [17].

**Fig. 2:**
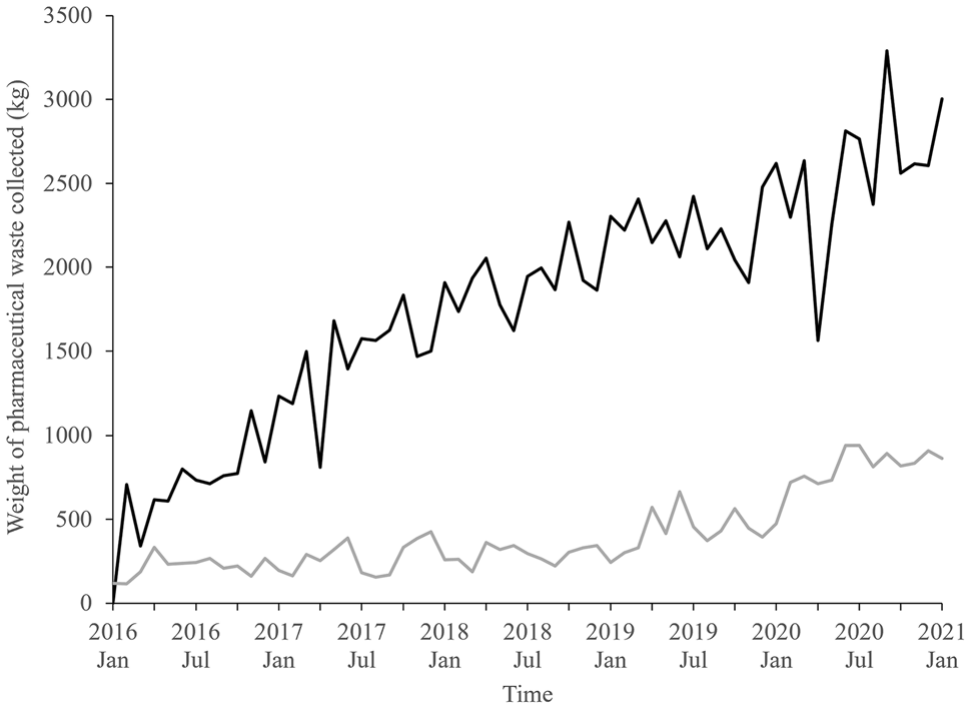
Changes in the total weight of pharmaceutical waste being produced in DHB-contracted pharmacies (black line) and hospitals (grey line) in Auckland by month between January 2016 and 2021

A roughly fourfold increase in the amount of pharmaceutical waste returned from community pharmacies was observed over the last 4 years, increasing from 759 kg produced in the month of September 2016 to 3290 kg in the month of September 2020 (Fig. 2). This increase was more pronounced in community pharmacies than hospital pharmacies. Hospital waste is separated into pharmaceutical waste (with cytotoxic waste entering a separate waste stream) and general medical waste. The proportion of pharmaceutical waste arising from hospitals was low, which could be because hospitals do not routinely receive unwanted medicines. It may reflect tight stock management, resulting in less expired medicines in this environment. It is also possible that hospitals incorrectly sort some pharmaceutical waste into a general medical waste stream. This might explain why, although hospitals are larger than community pharmacies and see a greater number of patients, community pharmacy waste made up 81.4% of all pharmaceutical waste generated by public hospitals and pharmacies in Auckland between January 2016 and December 2020. The number of pharmacies included in this data was 34% greater in 2020 compared to that in 2016, which could reflect an increase in the number of existing pharmacies that utilise the waste disposal service. In Auckland, the waste disposal service is funded by the three DHBs, so cost is unlikely to be a barrier to uptake; however, there may be an increased awareness of the service by both community pharmacies and their customers. Previous research has highlighted that accumulation of medicines in households is common, and that unwanted medicines are not always returned to pharmacies for disposal [6, 18, 19]. Further research is required to explore whether there have been changes in the disposal practices of unwanted medicines over this period, or if the increase in pharmaceutical waste could be attributed to changes in the prescription and consumption of medicines.

An increase in pharmaceutical waste from hospitals of 83.8% was recorded in 2020 compared with the weight of waste collected in 2019. This could be due to an increase in the use of disposable waste in hospital pharmacies amidst the COVID-19 pandemic, where more PPE contaminated with pharmaceuticals is entering the pharmaceutical waste stream. Similarly, an increase in medical waste has been reported in the other studies. In Barcelona (Spain), medical waste generation increased by 350% compared to the usual amount before the COVID-19 pandemic [20]. This medical waste included PPE such as face masks, overalls, and gloves, so the increased use and disposal of these items may be a major contributor. In China, it has been reported that the COVID-19 pandemic has increased medical waste generation from hospitals by sixfold [21]. This is reportedly also due to the overwhelming surge in PPE use. The present study focused on pharmaceutical waste and did not investigate general medical waste. A study based at Xanthi General Hospital in Greece over a 5 week period found that pharmaceutical waste comprised 3.9% w/w of the total hazardous medical waste produced by the hospital, which is a small fraction of medical waste produced in a hospital setting [22].

Only data from public hospitals and community pharmacies within the three Auckland DHBs that have a contract with Interwaste to provide waste disposal services were included in this data. Other sources of pharmaceutical waste such as private hospitals and clinics were not captured in this dataset. Furthermore, pharmaceutical waste from individual households are more likely to enter the general waste stream, as not many people return waste to pharmacies [6]. Pharmaceutical manufacturers and research institutes are likely to produce a significant amount of pharmaceutical waste compared to other healthcare activities, but are also not included in this dataset [23].

### Composition of pharmaceutical waste

During January 2021, 212 bins of pharmaceutical waste were collected by Interwaste from community pharmacies in the three Auckland DHBs, so we determined the composition of approximately 5.7% of bins collected in this period. During the 12 visits, 475 different products were identified, representing more than 75,000 units of medicine. Of these units of medicine, 30.5% were in blister packs (adherence aid packaging), 2.2% were loose in the bins, and the remaining 67.4% were packaged in pharmacy tablet bottles and skillets, or original manufacturer packaging. More than half of the pharmaceutical waste items were in the form of tablets (Table 2). Tablets and capsules alone made up 89% of all pharmaceutical waste items examined. This was not a surprise as tablets and capsules are the most common types of solid formulations prescribed [24]. Oral liquids only made up 0.12% of all pharmaceutical waste items, which may be because liquid dosage forms are more easily disposed of down the sink than solid dosage forms and therefore may be less likely to enter the pharmaceutical waste stream. It was not possible to accurately determine the quantity of liquid or semi-solid dosage forms in the audited waste, so one unit was defined as one original bottle or tube for liquids and semi-solids, whereas solid dosage forms were quantified as individual tablets or capsules. For this reason, the data may be skewed, because whereas one bottle of oral liquid may last for weeks or even a month, multiple tablets may be needed within a day; thus, the weighting of a “unit” between liquid formulations and solid formulations were different.

**Table 2:** Breakdown of all pharmaceutical waste items identified by dosage form. One unit represents one tablet, capsule, gum or device, or one original pack for liquids, topical formulations, drops, and sprays

Dosage form	Number of units	Portion of total composition (%)
Tablets	52,311	68.0
Capsules	18,888	24.6
Chewable tablets/gum	2115	2.7
Other^a^	2572	3.3
Medical devices	1039	1.4
Total	76,925	100

^a^Other includes dosage forms that made up less than 1% total units: powders, ampoules and injections, inhalers, lozenges, transdermal patches, topical formulations, suppositories, oral liquids, drops, enemas, and sprays

Medical devices made up 2% of all waste items found. These items included blood glucose strips, needles, and cleaning pads. As shown in Table 2, there were multiple dosage forms that only formed a small percentage of what was found. These were either less common dosage forms like suppositories, or inhalers and sprays, where one pack/device was considered a single unit.

#### Therapeutic classes of medicines

**Fig. 3:**
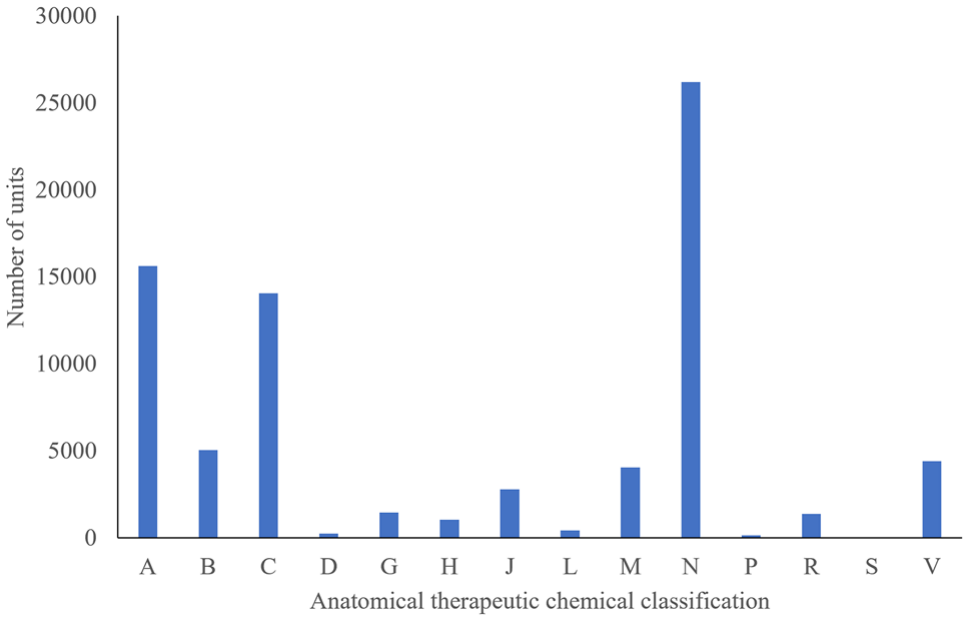
Distribution of the waste items into different therapeutic classes of drugs. **A** alimentary tract and metabolism, **B** blood and blood forming organs, **C** cardiovascular system, **D** dermatologicals, **G** genito urinary system and sex hormones, **H** systemic hormonal preparations, excluding sex hormones, **J** general antiinfectives for systemic use, **L** antineoplastic and immunomodulating agents, **M** musculoskeletal system, **N** nervous system, **P** antiparasitic products, insecticides and repellents, **R** respiratory system, **S** sensory organs, **V** various, including diagnostic agents and non-therapeutic products

Each pharmaceutical waste item identified in the audit was classified into therapeutic classes according to the ATC index [16], as shown in Fig. 3. The most common medicines were those acting on the nervous system, making up 34.1% of all waste items, followed by cardiovascular (18.3%) and alimentary tract and metabolism (20.3%). Nervous system drugs included antiepileptics such as gabapentin and sodium valproate, as well as both non-opioid and opioid analgesics such as paracetamol and tramadol. Cardiovascular drugs made up the second largest proportion of waste items found. These included diuretics such as furosemide, statins including atorvastatin and simvastatin, and beta blockers including metoprolol succinate. In a national population study looking at all registered patients in New Zealand at least 65 years old between 2010 and 2015, cardiovascular system drugs were the most widely prescribed, with alimentary tract and nervous system drugs also common [25]. Despite this age group only representing a fraction of the total population, they are the largest users of medicine and so it is not surprising to see that medicines more commonly prescribed in this age group are also appearing in the pharmaceutical waste stream. Similarly, an audit of medicines returned to pharmacies in the United Kingdom found that the prevalence of different medicines returned was related to prescribing patterns, with cardiovascular drugs the most commonly prescribed class of medicines as well as the most commonly returned medicines [26].

General antiinfective drugs for systemic use made up 3.6% of all pharmaceutical waste items. Of these, 93.1% were antibacterials such as flucloxacillin and amoxicillin, while the rest were antiprotozoals and antivirals. A previous study of municipal solid waste in Orange County, Florida found antimicrobial drugs present at high concentrations, especially ciprofloxacin and amoxicillin [27]. It is known that environmental contamination of antimicrobial drugs can lead to antimicrobial resistance [28]. It is unclear to what degree steam sterilisation via autoclaving, which is standard practice in New Zealand for the treatment of pharmaceutical waste items prior to landfilling, sufficiently deactivates drugs such as antimicrobials, and more research is needed to elucidate this.

The various category included dietary supplements, alternative medicines, and medical devices like blood glucose strips, needles, and swabs.

#### Common medicines identified

Table 3 lists the 20 medicines with the highest number of units identified during the audit. Gabapentin and paracetamol alone made up 12% of all pharmaceutical waste items found, and both appeared at high frequencies over the 12 visits. Tramadol hydrochloride, an opioid analgesic, appeared as the 26th most commonly found pharmaceutical waste item with 711 units found amongst 5 visits. Codeine phosphate and morphine sulphate ranked 46th and 69th, with 565 and 273 units found amongst 5 and 7 visits, respectively. Codeine and morphine are opioid analgesics, and are tightly regulated due to their potential for misuse, which can lead to opioid-related morbidity and mortality [29]. The ecotoxicological impacts of opioids in the environment such as their environmental fate and transformation are not fully known [30]. In New Zealand, codeine phosphate is a Class C2 controlled drug and morphine sulphate is a Class B1 controlled drug [31]. Clear documentation of Class A and B controlled drugs entering and leaving a pharmacy is required using a Controlled Drugs Register, including a dedicated page for controlled drugs disposal. It is not considered appropriate to dispose of controlled drugs in their original form. The Pharmaceutical Society of New Zealand recommends that controlled drugs are destroyed by breaking down any solid dosage forms into a powder and mixing with detergent to render them non-recoverable [32]. The inclusion of controlled drugs in the pharmaceutical waste stream in their original form suggests that this is not happening in all cases and that further education may be needed to highlight the appropriate process for disposing of controlled drugs.

**Table 3:** Top 20 most commonly identified drugs in pharmaceutical waste, in descending order of total number of units (tablet, capsule) and number of times each drug was identified over the 12 visits

Rank	Name	Units identified	Number of appearances
1	Gabapentin	4967	9
2	Paracetamol	4034	12
3	Omeprazole	2703	12
4	Docusate + Senna (Laxsol)	2665	10
5	Metformin	2266	9
6	Metoprolol succinate CR	1545	11
7	Aspirin EC	1410	9
8	Paracetamol + Codeine	1378	6
9	Ibuprofen	1368	9
10	Carbidopa + Levodopa ER	1214	4
11	Omega 3 Fish oil	1200	1
12	Warfarin sodium	1176	9
13	Furosemide	1123	12
14	Nicotine (Habitrol)	1070	2
15	Venlafaxine XR	1010	4
16	Atorvastatin	940	10
17	Felodipine ER	908	11
18	Flucloxacillin	887	8
19	Cilazapril	874	10
20	Quetiapine	852	9

#### Cytotoxic and teratogenic medicines were identified in pharmaceutical waste

Cytotoxic drugs pose a risk to human health when handled. In New Zealand, cytotoxic agents must be collected separately from pharmaceutical waste and destroyed by incineration. As incineration of pharmaceuticals is not permitted in New Zealand, this process involves shipping these drugs to be incinerated overseas [10]. Surprisingly, a total of 550 units of cytotoxic drugs were identified, representing 0.71% of all audited waste. The inclusion of cytotoxic waste in the pharmaceutical waste stream poses a risk to those handling the waste as well as possible ongoing effects to the environment.

Warfarin ranked number 12 by units found, appearing 9 times out of 12 visits. While not a cytotoxic drug, warfarin is a human teratogen, and has a high degree of acute oral toxicity [33]. It is also used as rodenticide. However, as toxicity requires the oral route, it is likely that warfarin will not accumulate to significant amounts to affect human, avian, or aquatic health [33]. Despite this, it is still recommended that warfarin should not enter the landfill like other general waste items [34].

The presence of cytotoxic and hazardous medicines in pharmaceutical waste highlights that further education and resources may be necessary to reduce these drugs entering the incorrect waste stream. However, the current practice of exporting cytotoxic waste offshore also requires review to maintain an appropriate disposal method that avoids the financial and environmental cost of international movement of hazardous waste [13]. While incineration in New Zealand instead of offshore would remove the costs of transport and shipping, a law change would be required to enable this. A more suitable improvement of current practices would be alternative technologies such as hydrothermal deconstruction, which has shown promise in degrading several pharmaceuticals to date, including local anaesthetics, antibiotics, and hormones [35–37]. If such a system could treat all pharmaceutical waste together in a single stream, that would remove challenges and inefficiencies around the separation of cytotoxic waste.

#### Expired and returned medicines

In terms of expiry, 29.8% of the waste items had not expired, 26.4% had expired, and the remaining 43.8% did not have an identifiable expiry date. Out of the medicines that were expired, it seemed that some were returned by patients, and others came from expired stock in the pharmacies. Many unexpired pharmaceutical items appeared to be unused. It is possible that these disposed medicines may have come from unwanted medicines that were returned by patients, so it would be unacceptable and unethical to re-use them. A previous study investigated reasons for medicines being returned by patients and found that the most common reason was because the prescriber had changed their medication regimen [38]. Another study investigated medicines returned to pharmacies during an 8 week period, where a total of 294 kg of medicines were returned [39].

#### Medicines with identifiable patient information

It was found that of items classed as medicines, 73% were labelled with identifiable patient information. This is in breach of Principle 2 of the Pharmacy Council Code of Ethics 2018 [40], which states that a pharmacist must safeguard and respect the confidentiality of patient information. Any technology developed to deconstruct pharmaceutical waste must be capable of destroying identifiable patient information.

In 2020, Interwaste collected an average of 193 bins of pharmaceutical waste from community pharmacies in Auckland per month. While the 12 bins investigated in this study represent only a fraction of this number, it gives an indication of the composition and breadth of pharmaceutical waste in New Zealand. There were instances where a single large sum of disposed medicines appeared to skew the data. For example, Omega 3 fish oil only appeared in one of the 12 visits, but it ranked the tenth most common item found. Similarly, nicotine replacement therapy was the 13th most common item, although it only appeared in two of the visits. However, these data give us an indication of the distribution of the types of pharmaceutical items found. New Zealand is increasingly generating high amounts of pharmaceutical waste, and the current waste treatment procedures mean these wastes end in landfills and eventually the environment. More research into the efficacy of autoclaving pharmaceutical waste is needed and alternative options for safe pharmaceutical waste treatment and disposal that avoid landfilling are required.

## Conclusion

Pharmaceutical waste production in New Zealand is increasing, with waste collected by community and hospital pharmacies in Auckland increasing more than fourfold from 2016 to 2020. A snapshot of the types of waste found in pharmaceutical waste bins from community pharmacies in Auckland identified gabapentin and paracetamol as the most common drugs, together making up 12% of all pharmaceutical waste items examined. Over the 12 visits, 475 different pharmaceutical products were identified, which highlights the breadth of drugs in this waste stream. A range of dosage forms and hence materials were identified, which could present challenges for future waste treatment approaches. Potentially hazardous drugs such as warfarin were identified, alongside the surprising finding of cytotoxic drugs. This raises concerns regarding ongoing environmental effects when these drugs enter landfill, as well as more immediate risks to individuals involved in the processing of pharmaceutical waste. Drugs with high potential for misuse including morphine were identified, as well as antimicrobial drugs, which could augment antimicrobial resistance. More research is needed to determine whether current practices for the management of pharmaceutical waste are suitable for the wide range of drugs identified. It is likely that alternative options for the safe treatment and disposal of pharmaceutical waste are required.
